# Epidemiology and prognostic analysis of patients with pancreatic signet ring cell carcinoma: a population-based study

**DOI:** 10.1186/s12876-022-02543-z

**Published:** 2022-11-16

**Authors:** Duorui Nie, Qingxia Lan, Yue Huang, Chongkai Fang, Yang Cao, Yao Chen

**Affiliations:** 1 Graduate School, Hunan University of Chinese Medicine, Changsha, 410208 Hunan Province China; 2 Department of Oncology, The First Affiliated Hospital of Guangzhou University of Chinese Medicine, Guangzhou, 510405 Guangdong Province China; 3 First Clinical Medical College, Guangzhou University of Chinese Medicine, Guangzhou, 510405 Guangdong Province China

**Keywords:** Epidemiology, Pancreatic signet ring cell cancer, SEER database, Survival analysis

## Abstract

**Background:**

Pancreatic signet ring cell carcinoma (PSRCC) is a rare tumour subtype with poorly understood epidemiological characteristics and prognosis. We attempted to comprehensively characterise the epidemiology and survival outcomes of PSRCC.

**Methods:**

Patients diagnosed with PSRCC between 2000 and 2018 were identified using Surveillance, Epidemiology and End Results Stat 8.3.9.2 software. Age-adjusted incidence and survival were calculated. Survival curves were plotted using the Kaplan–Meier method, and the differences between survival curves were compared using the log-rank test. Cox proportional hazards models were used to evaluate factors that independently predict overall survival. The primary analysis was a complete case analysis; multiple imputations were employed in a sensitivity analysis.

**Results:**

We identified 585 eligible patients with PSRCC. The overall annual incidence from 2000 to 2018 was 0.349 (95% CI, 0.321–0.379) per million population. The incidence increased significantly in patients over 55 years of age and peaked at about 80 years of age (2.12 per million). Males and Black patients had the highest incidence. The observed survival rates at 1, 2 and 5 years were 20.1, 8.3 and 3.4%, respectively. Survival analysis revealed that primary surgery and chemotherapy are effective treatments for patients with PSRCC (*P* < 0.05). According to multivariate Cox regression analysis, early stage and receiving surgery and chemotherapy were favourable factors (*P* < 0.05). Similar conclusions were drawn from the interpolated data.

**Conclusions:**

PSRCC is a highly malignant tumour that predominates in elderly, male and Black patients. The prognosis is poor with a 5-year survival rate of 3.4%; however, multivariate analysis and adjusted models accounting for missing data revealed that early diagnosis, surgery and chemotherapy are effective in improving the prognosis.

**Supplementary Information:**

The online version contains supplementary material available at 10.1186/s12876-022-02543-z.

## Introduction

Signet ring cell carcinoma (SRCC) is a rare histological type of adenocarcinomas in which the nucleus is squeezed sideways to form a “signet ring” appearance owing to the intra-cytoplasmic mucin vacuoles containing more than 50% of the mucin protein [1]. Over 96% of SRCC cases originate in the stomach, with the remaining cases originating in the rectum, gallbladder, colon, breast, bladder and pancreas [2]. Pancreatic signet ring cell carcinoma (PSRCC) is a very rare tumour occurring in less than 1% of pancreatic carcinoma [3]. Diagnosis of primary PSRCC requires a tumour with at least 50% signet ring cell morphology and the exclusion of metastases from other sites [1, 4]. Patients with pancreatic cancer without obvious clinical symptoms are frequently not diagnosed until the tumour is at an advanced stage. This applies to PSRCC, which has a reported first-visit metastasis rate of 69.4%, contributing to its poor prognosis. Furthermore, the ease of invasion of surrounding tissues and metastasis contribute to the poor prognosis [5]. In addition to these properties of PSRCC, no standard therapy has been established. The value of chemotherapy has been debated. Although SRCC is generally considered to be insensitive to chemotherapy, Radojkovic et al. [6] reported a case of a patient with PSRCC who responded effectively to gemcitabine neoadjuvant chemotherapy. Furthermore, there is controversy surrounding the benefits of surgery and radiation.

Owing to the rare nature of PSRCC, it hinders our understanding of the clinical features and treatment of PSRCC. To our knowledge, three large studies of PSRCC were based on data from the US Surveillance, Epidemiology and End Results (SEER) registry. Patel reported the epidemiological factors and treatment effects on 497 patients with PSRCC diagnosed between 1985 and 2013 [7]. Although this study is the most detailed description of PSRCC, it did not exclude patients with non-pathological diagnoses, and it also lacked information about chemotherapy and failed to measure its value. Furthermore, the impact of surgery and radiotherapy in each stage was also unknown, and the study did not describe the age-adjusted incidence of PSRCC. The other two studies involving PSRCC evaluated the effect of primary tumour location on survival of SRCC [8] and reported the trends of incidence and survival in patients with gastroenteropancreatic [9]. Although both studies included some of the clinical features of PSRCC, a detailed description or analysis of its incidence, characteristics, prognostic factors and treatment methods were not provided. Importantly, the role of chemotherapy has not been elucidated.

Therefore, to describe the clinical characteristics of PSRCC in more detail, we conducted this study: (1) to determine the epidemiological characteristics of the patients, such as determining a more accurate age-adjusted incidence and calculating age-adjusted mortality for the first time (2) to clarify the prognostic impact of surgery, radiotherapy and chemotherapy in each summary stage and (3) to ascertain the prognostic impact of chemotherapy on overall survival (OS).

## Patients and methods

### Study population

The SEER database collects information on tumour clinicopathological, treatment and vital status from population-based cancer registries covering approximately 27.8% of the U.S. population (https://seer.cancer.gov). This study does not require Institutional Review Board approval since the SEER study data is available to the public, and we have access to the study data under a SEER license (Login number: 13487-NOV2020). Furthermore, we declared all methods were performed per relevant guidelines and regulations.

“The SEER Research Plus Data, 18 Registries, Nov 2020 Sub (2000–2018)” was used to calculate the incidence, demographic and clinicopathologic characteristics and survival. Patients with only one primary PSRCC diagnosed between 2000 and 2018 by pathologically confirmation were selected. All populations were used for incidence calculations; however, when survival analysis was performed those with unknown follow-up time and age were not included. The cohort for survival analysis included 585 patients with PSRCC who met the inclusion criteria.

### Definition of variables

The following variables were extracted from the SEER database: age at diagnosis (≤67 and > 67), gender (male and female), marital status (married or unmarried and unknown), race (White, Black and others [American Indian/AK Native and Asian/Pacific Islander]), year of diagnosis (2000–2009 and 2010–2018), regional nodes positive (negative, positive and no nodes examined), tumour size (≤4 cm and > 4 cm), primary tumour location (head, body, tail and other), summary stage (localised, regional and distant), differentiation grade (I/II and III/IV), primary surgery (yes or no), radiation (yes or no/unknown), chemotherapy (yes or no/unknown), vital status and survival months. Age at diagnosis was converted to binary data by using a median cut-off value of 67. American Indian/AK Native and Asian/Pacific Islander were classified as “Others”. “C25.3-Pancreatic Duct”, “C25.7-Other specified parts of Pancreas” and “C25.4-Islets of Langerhans” were classified as “Others”. Consequently, the primary sites were grouped by “Head”, “Body”, “Tail”, “Other” and “non-otherwise specified (NOS)”. The staging data were based on the SEER Summary Stage 2000, which categorises the disease’s extent as “localised”, “regional” or “distant” [10]. A localised disease is defined as a tumour localised to the pancreas. The regional disease involves tumours of the pancreas extending to adjacent organs, regional lymph nodes or both. A distant disease is defined as those who had distant metastases at the time of diagnosis.

Survival months were defined as the length of time from the month of initial diagnosis to the patient’s death from any cause or the last month of follow-up (November 2020). The primary outcome was OS, which was defined as an all-cause of death. Furthermore, survival rates were estimated using the observed survival, expected and relative survival rates with age adjustment [11, 12]. Expected survival rates are calculated for individual years relative to the standard population of the United States (U.S.) 1992–2006. Relative survival was defined as observed survival as a percentage of expected survival.

### Statistics analysis

Using SEER*Stat software, age-adjusted incidence trends were estimated and adjusted for the 2000 U.S. standard population, as well as observed, relative and expected survival. Classification variables were expressed as frequency and percentage, whereas continuity variables were expressed as quartiles. The Kaplan–Meier method was used to plot survival curves, and the log-rank test was performed to assess the differences between the survival curves. Univariate and subsequent multivariate Cox regression analyses were performed to evaluate factors that have independent predictive effects on the OS and calculate hazard ratios (HRs) and 95% confidence intervals (CIs).

We used multiple imputation (MI) replicates adjusted for Cox analysis to perform a sensitivity analysis since our dataset contained missing values [13, 14]. The original data, as they were retrieved from the database, were introduced as the first model was built. Following imputation analysis, the pooled data were introduced in a new Cox regression model using the same covariates. Statistical analysis was performed with R 4.0.4 (http://www.r-project.org/) and GraphPad Prism version 8.0.0 for Windows (GraphPad Software, San Diego, California USA, www.graphpad.com), and *P* < 0.05 was considered statistically significant.

## Results

### Demographic and clinicopathologic characteristics

In this study, a total of 585 eligible patients with PSRCC were identified. The clinical features of the patients are presented in Table 1. The median age was 67 years. The male-to-female ratio in this study was 1.25:1, with 325 (55.6%) male patients and 260 (44.4%) female patients. White patients account for 82.7% of the population. Of 585 patients, 383 (65.5%) patients had a record of available tumour size. Among the known records, 43.6% of the tumours were > 4 cm and 56.4% were ≤ 4 cm. The most common grade of differentiation was III/IV, accounting for 87.9%, while grade I/II accounted for 12.1%.

**Table 1:** The clinical features of the patients with pancreatic signet ring cell carcinoma (PSRCC)

Variables	Available data-N (%)	N (%)
**Age at diagnosis**	585 (100)	
**67.0 [59.0, 75.0]**		
< =67		300 (51.3)
> 67		285 (48.7)
**Sex**		
Female	585 (100)	260 (44.4)
Male		325 (55.6)
**Race**	583 (99.7)	
White		482 (82.7)
Black		62 (10.6)
Other		39 (6.7)
**Year of diagnosis**	585 (100)	
2000–2009		323 (55.2)
2010–2018		262 (44.8)
**Marital status at diagnosis**	560 (95.7)	
Married		331 (59.1)
Unmarried		229 (40.9)
**Differentiation grade**	280 (47.9)	
I/II		34 (12.1)
III/IV		246 (87.9)
**Tumor size**	383 (65.5)	
< =4		216 (56.4)
> 4		167 (43.6)
**Primary site**	462 (79.0)	
Head		273 (46.7)
Body		60 (10.3)
Tail		85 (14.5)
Other		44 (7.5)
**Regional nodes status**	578 (98.8)	
Negative		41 (7.1)
Positive		88 (15.2)
No nodes examined		449 (77.7)
**Summary stage**	566 (96.8)	
Localized/Regional		170 (30.0)
Distant		396 (70.0)
**Surgery**	581 (99.3)	
Yes		100 (17.2)
No		481 (82.8)
**Chemotherapy**	585 (100)	
Yes		263 (45.0)
No/Unknown		322 (55.0)
**Radiation**	585 (100)	
Yes		80 (13.7)
No/Unknown		505 (86.3)

At diagnosis, 70% of patients had distant metastatic disease. Chemotherapy, which accounts for 45% of PSRCC treatment, is the initial treatment option for patients. One hundred (17.2%) patients underwent surgery, while 80 (13.7%) patients received radiation therapy. When presented in summary stages, details of treatment are presented in Table 2. Of 170 patients with localised/regional disease, 78 (45.9%) underwent primary surgery, 87 (51.2%) received chemotherapy and 54 (31.8%) received radiotherapy.

**Table 2:** The clinical features of the patients with pancreatic signet ring cell carcinoma (PSRCC) according to stratification by summary stage

Therapy	Levels	Distant	Localised/Regional
Surgery (%)	No	374 (94.7)	92 (54.1)
	Yes	21 (5.3)	78 (45.9)
Chemotherapy (%)	No/Unknown	224 (56.6)	83 (48.8)
	Yes	172 (43.4)	87 (51.2)
Radiation (%)	No/Unknown	371 (93.7)	116 (68.2)
	Yes	25 (6.3)	54 (31.8)

### Age-adjusted incidence

**Fig. 1:**
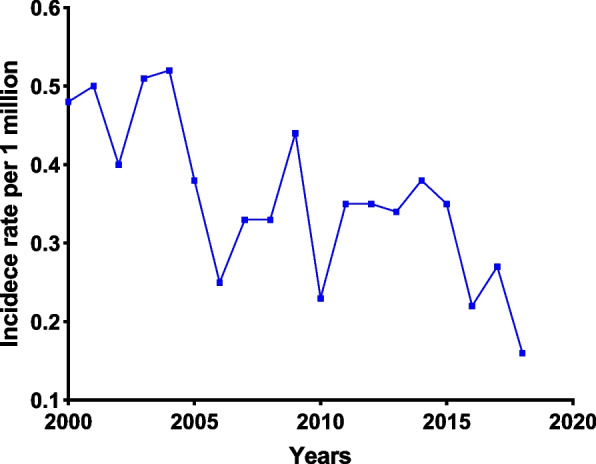
The age-adjusted incidence of pancreatic signet ring cell carcinoma (PSRCC) from 2000 to 2018

**Fig. 2:**
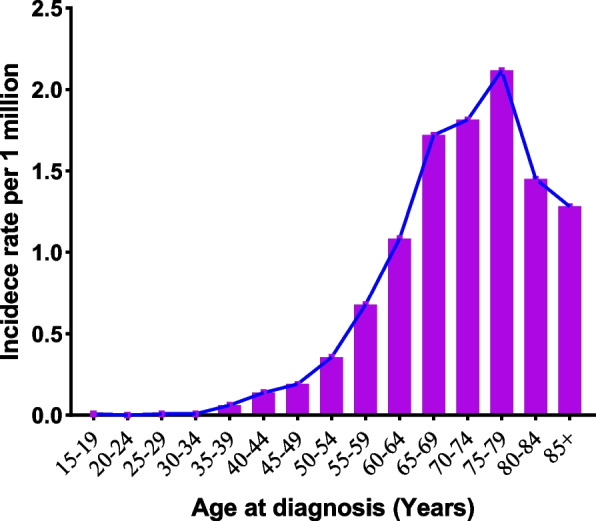
The age-adjusted incidence rate of pancreatic signet ring cell carcinoma (PSRCC) in different age groups

**Fig. 3:**
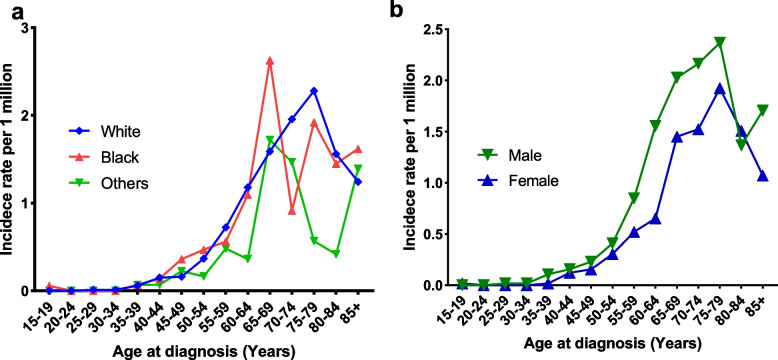
The age-stratified incidence rates for different races and genders

Table 3 summarises the age-adjusted incidence rates for common clinical features. The overall annual incidence of PSRCC within the SEER-18 database was 0.349 (95% CI, 0.321–0.379) per million population between 2000 and 2018. The age-adjusted incidence rates are higher in men than in women, higher in Black patients than in other races and tumours in the head of the pancreas are more common between 2000 and 2009. As presented in Fig. 1, the annual adjusted incidence rate decreased with time, from 0.48 per million in 2000 to 0.16 per million in 2018. Over the past few decades, there has been a significant decline in the incidence of PSRCC. The incidence of PSRCC increased significantly with age, especially in patients aged 55 and above, and gradually reduced after 70 years of age, peaked between the ages 75 and 79 years (2.12 per million) and gradually declined thereafter (Fig. 2). Similar results were observed when the incidence was grouped by sex (Fig. 3a). The changes in incidence rates by age across ethnic groups are presented in Fig. 3b. The incidence peaked earlier in Black patients than in White patients, with the first peak occurring between the ages of 65 and 69 for Black patients in a bimodal distribution and between the ages 75 and 79 for White patients. The incidence rate of Black patients was higher than that of other races (0.369 per million).

**Table 3:** The age-adjusted incidence of pancreatic signet ring cell carcinoma (PSRCC) in Surveillance, Epidemiology and End Results (SEER) 2000–2018

Variables	Incidence^a^	95% CI
**Overall**	0.349	0.321–0.379
**Age at diagnosis**		
15–19	0.009	0–0.049
20–24	0.000	0–0.032
25–29	0.009	0–0.049
30–34	0.009	0–0.049
35–39	0.062	0.025–0.127
40–44	0.139	0.079–0.225
45–49	0.192	0.102–0.290
50–54	0.356	0.253–0.487
55–59	0.680	0.526–0.865
60–64	1.085	0.868–1.340
65–69	1.720	1.410–2.079
70–74	1.814	1.453–2.237
75–79	2.116	1.675–2.637
80–84	1.451	1.037–1.976
85+	1.282	0.888–1.792
**Sex**		
Male	0.420	0.375–0.470
Female	0.287	0.253–0.325
**Race**		
White	0.359	0.328–0.393
Black	0.369	0.280–0.475
Other	0.220	0.057–0.569
**Year of diagnosis**		
2000–2009	0.409	0.366–0.457
2010–2018	0.293	0.258–0.331
**Primary site**		
Head	0.162	0.144–0.183
Body	0.037	0.028–0.047
Tail	0.050	0.040–0.062
Other	0.026	0.019–0.035

^a^Incidence rates are calculated per million population

### Survival analysis

Subsequently, the age-adjusted survival rate was calculated using SEER*Stat software version 8.3.9 and is presented in Table 4. The 1-, 2- and 5-year observed survival rates were 20.1, 8.3 and 3.4%, respectively.

**Table 4:** Observed, expected and relative survival rates of pancreatic signet ring cell carcinoma (PSRCC) patients from 2000 to 2018

Years	Observed survival (SE)	Expected survival	Relative survival (SE)
1	20.1% (1.7%)	97.50%	20.5% (1.7%)
2	8.30% (1.2%)	94.90%	8.70% (1.2%)
3	5.30% (1.0%)	92.40%	5.60% (1.0%)
4	3.80% (0.8%)	89.50%	4.10% (0.9%)
5	3.40% (0.8%)	86.60%	3.70% (0.9%)

**Fig. 4:**
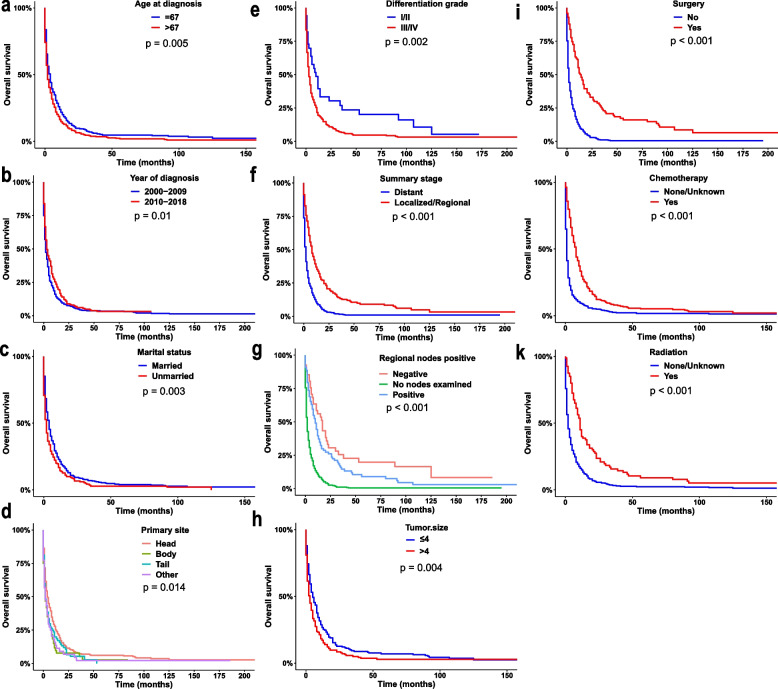
Kaplan–Meier survival curves with statistically significant clinical features in patients with pancreatic signet ring cell carcinoma (PSRCC). age at diagnosis (**a**), year of diagnosis (**b**), marital status (**c**), primary site (**d**), differentiation grade (**e**), summary stage (**f**), regional nodes status (**g**), tumour size (**h**), surgery (**i**), chemotherapy (**j**), radiation (**k**)

**Fig. 5:**
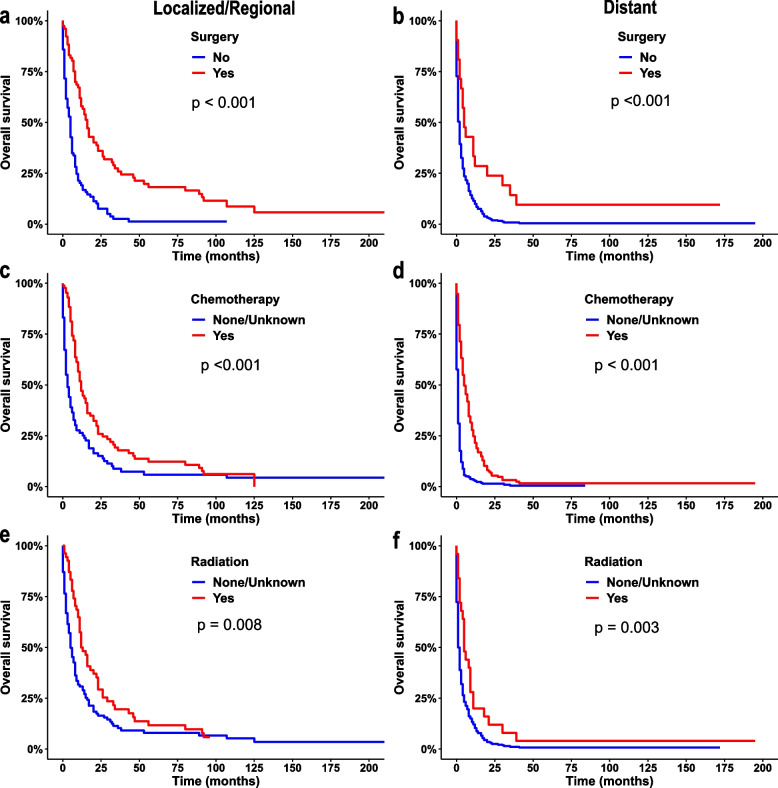
Kaplan–Meier survival curves for patients with pancreatic signet ring cell carcinoma (PSRCC) based on the summary stage. **a**, **c**, **e**: based on localised/regional stage, **a**: surgery, **c**: chemotherapy, **e**: radiation (**b**, **d**, **f**): based on distant stage, **b**: surgery, **d**: chemotherapy, **f**: radiation

The log-rank test revealed that there may be a relationship between OS and the variables age at diagnosis (Fig. 4a), year of diagnosis (Fig. 4b), marital status (Fig. 4c), primary site (Fig. 4d), differentiation grade (Fig. 4e), summary stage (Fig. 4f), regional nodes status (Fig. 4g), tumour size (Fig. 4h), surgery (Fig. 4i), chemotherapy (Fig. 4j) and radiation (Fig. 4k). The stage has a great impact on the course of treatment. Therefore, the effectiveness of treatment in different summary stages was compared. The Kaplan–Meier survival curve stratified according to the therapy and summary stage are displayed in Fig. 5. Patients who underwent primary tumour surgery, chemotherapy and radiation had substantially improved OS (Fig. 5, *P* < 0.05).

Specifically, compared with the reference group in univariate Cox analysis (Table 5), patients with older age (HR, 1.27; 95% CI, 1.07–1.50; *P* = 0.005), unmarried (HR, 1.31; 95% CI, 1.10–1.56; *P* = 0.002), grade III/IV disease (HR, 1.84; 95% CI, 1.25–2.72; *P* = 0.002), tumours not at the head of the pancreas (Body: HR, 1.47; 95% CI, 1.10–1.97; *P* = 0.009, Tail: HR, 1.29; 95% CI, 1.00–1.66; *P* = 0.048, Other: HR, 1.39; 95% CI, 1.01–1.93; *P* = 0.045) and larger tumour size (HR, 1.37; 95% CI, 1.11–1.69; *P* = 0.003) were associated with poor prognosis (HR > 1, *P* < 0.05) whereas patients with earlier stage (summary stage: localised/regional, HR, 0.44; 95% CI, 0.36–0.53; *P* < 0.001; regional nodes status: negative, HR, 0.71; 95% CI, 0.48–1.06; *P* = 0.097, no nodes examined, HR, 2.53; 95% CI, 1.98–3.24; *P* < 0.001) or treated with radiotherapy (HR, 0.45; 95% CI, 0.35–0.58; *P* < 0.001), chemotherapy (HR, 0.41; 95% CI, 0.35–0.49; *P* < 0.001) and surgery (HR, 0.31; 95% CI, 0.24–0.40; *P* < 0.001) were associated with favourable prognosis (HR < 1, *P* < 0.05). When these factors were incorporated in multivariate Cox regression, it was indicated that the summary stage, regional nodes status, surgery and chemotherapy were independent factors for patients with PSRCC.

**Table 5:** Univariate and multivariate regression analysis of overall survival (OS) in patients with pancreatic signet ring cell carcinoma (PSRCC)

Variables	Levels	Univariate	Multivariate		
		HR (95% CI)	***P***-value	HR (95% CI)	***P***-value
**Age at diagnosis**	<=67	1		1	
	> 67	1.27 (1.07–1.50)	**0.005**	1.22 (0.87–1.71)	0.244
**Sex**	Female	1			
	Male	1 (0.84–1.18)	0.982	/	/
**Race**	Black				
	Other	1.15 (0.76–1.73)	0.507	/	/
	White	0.93 (0.71–1.23)	0.616	/	/
**Year of diagnosis**	2000–2009	1		1	
	2010–2018	0.80 (0.67–0.95)	**0.009**	0.71 (0.50–1.00)	0.052
**Marital status at diagnosis**	Married	1		1	
	Unmarried	1.31 (1.10–1.56)	**0.002**	1.32 (0.93–1.86)	0.120
**Differentiation grade**	I/II	1		1	
	III/IV	1.84 (1.25–2.72)	**0.002**	1.41 (0.85–2.35)	0.183
**Tumour size**	<=4	1		1	
	> 4	1.37 (1.11–1.69)	**0.003**	1.02 (0.72–1.45)	0.913
**Primary site**	Head	1		1	
	Body	1.47 (1.10–1.97)	**0.009**	1.38 (0.78–2.43)	0.272
	Tail	1.29 (1.00–1.66)	**0.048**	1.45 (0.88–2.38)	0.140
	Other	1.39 (1.01–1.93)	**0.045**	1.27 (0.72–2.22)	0.412
**Regional nodes status**	Positive	1		1	
	Negative	0.71 (0.48–1.06)	0.097	0.58 (0.35–0.95)	**0.031**
	No nodes examined	2.53 (1.98–3.24)	**< 0.001**	0.74 (0.35–1.57)	0.440
**Summary stage**	Distant	1		1	
	Localised/Regional	0.44 (0.36–0.53)	**< 0.001**	0.66 (0.45–0.97)	**0.034**
**Surgery**	No	1		1	
	Yes	0.31 (0.24–0.40)	**< 0.001**	0.30 (0.14–0.65)	**0.002**
**Chemotherapy**	No/Unknown	1		1	
	Yes	0.41 (0.35–0.49)	**< 0.001**	0.42 (0.28–0.63)	**< 0.001**
**Radiation**	No/Unknown	1		1	
	Yes	0.45 (0.35–0.58)	**< 0.001**	1.07 (0.66–1.74)	0.782

Statistically significant variables are highlighted in bold

### Multiple imputation (MI) and sensitivity analysis

MI procedures were applied to account for missing data of some key variables in our study, and the results are presented in supplementary Table 1. Receiving surgery (HR, 0.40; 95% CI, 0.26–0.61; *P* < 0.001), chemotherapy (HR, 0.36; 95% CI, 0.30–0.44; *P* < 0.001, and an early stage at diagnosis (summary stage: localised/regional, HR, 0.62; 95% CI, 0.49–0.79; *P* < 0.001; regional nodes status: negative, HR, 0.65; 95% CI, 0.42–0.99; *P* = 0.046) remained associated with a better prognosis in multivariate Cox regression following MI. The most recent year of diagnosis (HR, 0.741; 95% CI, 0.619–0.886; *P* < 0.001) was also associated with a better prognosis, while unmarried patients (HR, 1.322; 95% CI, 1.092–1.601; *P* = 0.005) and older years (HR, 0.74; 95% CI, 0.62–0.89; *P* = 0.048) were associated with a worse prognosis.

## Discussion

Due to its rarity, the studies on PSRCC were case reports, and three large studies were based on SEER registries. The published literature on PSRCC is reviewed in Table 6. Three of them, Wu et al. [8], Li et al. [9] and Patel et al. [7] described the clinical characteristics of PSRCC. Overall, our study provides comprehensive information on the incidence, clinical features, treatment and prognosis of patients with PSRCC.

**Table 6:** Summary of studies on pancreatic signet ring cell carcinoma (PSRCC)

First Auth	Year of publication	Data Source	Years of diagnosis	N	Male (%)	Mean Age
Sangang W	2017	SEER database	1988–2012	441	232 (52.6)	/
Hui Li	2019	SEER database	2000–2014	593	330 (55.6%)	67.4
Mausam Patel [7]	2018	SEER database	1985–2013	497	271 (54.5)	66.6
Tracey [7], Chow [15], McArthur [16], Marcy [17], Terada [18], Karaahmet [19], Nauta [20], Radojkovic [6], Yepuri [21], Alexander [3], Campbell [22]	1984-2020	Case reports	1984–2020	11	6 (54.5)	66.8

The age-adjusted annual incidence of PSRCC was 0.349 per million population in the U.S. According to Li et al. [9], between 2000 and 2014, the age-adjusted incidence of PSRCC marginally decreased. Similar findings were observed in our study; however, we described the incidence of PSRCC in more detail. The incidence decreased from 0.48 to 0.16 per million between 2000 to 2018. Although the reasons for the decline are unclear, we speculate that it may be owing to the advancements in diagnostic techniques that can more accurately distinguish tumours similar to PSRCC [18, 23], resulting in a decrease in the incidence of PSRCC. Moreover, we observed that the incidence of PSRCC increased significantly in patients over 55 years of age, gradually decreased after 70 years of age, peaked at about 80 years of age (2.12 per million) and gradually decreased thereafter. Therefore, the differential diagnosis of PSRCC in individuals over 55 years of age requires greater focus. In addition, the peak age of onset varies amongst ethnic groups. It suggests that different ethnic groups have different concerns.

The demographics of PSRCC in the U.S. described in this study are comparable to those previously reported, with the average age reported in most SEER-based studies being approximately 67 years old. All studies reported male prevalence of 52.6–55.6%, and in our study, the male prevalence in PSRCC was 55.6%. Compared with the other three studies, we additional reported the marital status of PSRCC and observed that most patients were married. The addition of lymph node metastasis and tumour size enhanced our understanding of the clinicopathological features of PSRCC.

Age, primary site, grade, summary stage and local treatment have been previously demonstrated to be independent prognostic factors of OS in univariate Cox regression analysis [7]. However, previous studies lacked crucial information, such as marital status, regional nodes status and chemotherapy, and failed to assess their impact on OS. Our study indicated that chemotherapy can also significantly affect OS. Subsequent multivariate Cox analysis revealed that summary stage, regional nodes status, surgery and chemotherapy can significantly affect OS in both complete case and MI analyses. In addition, the stage at diagnosis is significantly correlated with prognosis, and the late stage is associated with poor prognosis, suggesting that early and timely diagnosis is also an effective measure to improve the prognosis of patients.

PSRCC is a rare disease, thus, limited investigations have been performed on it. Clinically, the therapy method of PSRCC is mostly referred to as pancreatic ductal adenocarcinoma. Surgery is usually recommended for non-metastatic PSRCC, while chemotherapy is the primary treatment option for metastatic PSRCC. In this study, the survival curve of the treatment mode was stratified based on the summary stage. We observed that surgery for primary tumours not only significantly improved OS at localised/regional stages but also distant stages. Indeed, tumour resection remains the only way to cure and is usually the preferred treatment for resectable or locally advanced pancreatic tumours [19]. However, it remains unclear how surgery can affect PSRCC metastases. Previous studies have shown that primary surgery can improve the OS of patients with metastatic pancreatic cancer [24, 25]. As a variant of adenocarcinoma, surgery may be a potential treatment option for patients with metastatic PSRCC, and this possibility is even greater owing to improvements in neoadjuvant chemotherapy [6].

Similarly, previous research lacked information about chemotherapy and failed to assess the value of chemotherapy for PSRCC. Our findings disprove the preconceived notion that gastric SRCC types are insensitive to chemotherapy and demonstrate that chemotherapy is an independent prognostic factor [26]. These findings further illustrate the importance of chemotherapy in the prognosis of PSRCC. To our surprise, radiotherapy is not an independent predictor of OS, and it was unclear whether it is due to the statistical failure to meet the criterion imposed by the sample size limit or caused by a special entity. However, as a local treatment method, radiotherapy has been proven to improve local control [27].

In sensitivity analysis, unmarried patients had a worse prognosis. The important role of marital status in pancreatic cancer is increasingly recognised [28–30] and has also been reported in subtypes of pancreatic ductal adenocarcinoma [31, 32]. However, the underlying mechanisms associated with marital status and survival can be multiple and complex, it may be associated with positive facilitation in marriage, such as better health habits, psychosocial support and greater financial resources. Older age was also associated with a worse prognosis. In addition, the most recent year of diagnosis was associated with a better prognosis. Since there have been advances over time regarding diagnostic and therapeutic approaches, especially the popularisation of multidrug chemotherapy regimens in 2011, which has improved the prognosis [33, 34]. The upper limit of the proportion of tumours that may be accurately estimated using this technique is reached when estimating the tumour grade, tumour size and primary tumour location. Therefore, rather than performing a primary analysis, a sensitivity analysis using MI data was performed. Inconsistencies in the interpolated data need to be interpreted with caution.

Although the SEER database provides us with a larger cohort to study the clinical characteristics of PSRCC, some limitations are also worth noting. Firstly, our study is a retrospective study, which may have selection bias and recall bias. Secondly, some valuable information is limited, such as the patient’s physical condition, comorbidities and specific treatment information, making the impact of these variables on survival difficult to assess and control. In addition, although the efficacy of chemotherapy was well validated, patients with unknown chemotherapy data were assigned to the “no/unknown” category, which in reality included those who received chemotherapy. Therefore, the trends we report may understate the actual rates due to misclassification. Finally, although we performed MI on the dataset and performed a master analysis and sensitivity validation of our analysis, discarding cases with missing values or performing separate analyses for cases with and without a complete dataset would have introduced a strong selection bias. Hence, to verify our findings in the future, more comprehensive data will be required.

In conclusion, PSRCC is a highly malignant tumour that predominates in elderly, male and Black patients. Approximately 70% of patients with PSRCC had distant metastasis at diagnosis, and the overall prognosis was poor. The 1-, 2- and 5-year observed survival rates were only 3.4, 8.3 and 20.1%, respectively. Timely diagnosis, surgery and chemotherapy are effective at improving the prognosis.

## Supplementary Information

**Additional file 1: Supplementary Table 1**. Multivariate regression analysis of OS in patients with PSRCC after multiple interpolation.

## Data Availability

Data for this study are available from the Surveillance Research Program, National Cancer Institute SEER*Stat software (www.seer.cancer.gov/seerstat) version 8.4.0. Detailed steps can be obtained by referring to the inclusion and exclusion criteria in the methodology.
