# Supplementary material for: Epidemiology and prognostic analysis of patients with pancreatic signet ring cell carcinoma: a population-based study
Source: BMC Gastroenterol. 2022 Nov 16;22:458. doi: 10.1186/s12876-022-02543-z (PMC9667582; doi:10.1186/s12876-022-02543-z)
Supplement: Supplementary file 1 — Additional file 1: Supplementary Table 1. Multivariate regression analysis of OS in patients with PSRCC after multiple interpolation. [file 12876_2022_2543_MOESM1_ESM.docx]

Supplementary table 1 Multivariate regression analysis of OS in patients with PSRCC after multiple interpolation

| **Variables** | **Levels** | **Multivariate** | |
| --- | --- | --- | --- |
|  |  | **HR (95%CI)** | **P-value** |
| Age at diagnosis | <=67 |  |  |
|  | >67 | 1.20 (1.00-1.42) | **0.048** |
| Year of diagnosis | 2000-2009 |  |  |
|  | 2010-2018 | 0.74 (0.62-0.89) | **0.002** |
| Marital status at diagnosis | Married |  |  |
|  | Unmarried | 1.33 (1.08-1.65) | **0.012** |
| Differentiation grade | I/II |  |  |
|  | III/IV | 1.17 (0.86-1.61) | 0.332 |
| Tumor size | <=4 |  |  |
|  | >4 | 1.18 (0.94-1.47) | 0.160 |
| Primary site | Head |  |  |
|  | Body | 1.33 (0.99-1.78) | 0.063 |
|  | Tail | 1.07 (0.80-1.42) | 0.658 |
|  | Other | 1.31 (0.95-1.82) | 0.104 |
| Regional nodes status | Positive |  |  |
|  | Negative | 0.65 (0.42-0.99) | **0.046** |
|  | No nodes examined | 1.06 (0.73-1.53) | 0.775 |
| Summary stage | Distant |  |  |
|  | Localized/Regional | 0.62 (0.49-0.79) | **<0.001** |
| Surgery | No |  |  |
|  | Yes | 0.40 (0.26-0.61) | **<0.001** |
| Chemotherapy | No/Unknown |  |  |
|  | Yes | 0.36 (0.30-0.44) | **<0.001** |
| Radiation | No/Unknown |  |  |
|  | Yes | 0.87 (0.66-1.15) | 0.328 |
|  |  |  |  |

**Statistically significant variables are highlighted in bold**
